# A Comprehensive Two-Decade Analysis of Lymphoma Incidence Patterns in Saudi Arabia

**DOI:** 10.3390/jcm13061652

**Published:** 2024-03-13

**Authors:** Ahmed M. Basudan, Mohammed Althani, Manal Abudawood, Raed Farzan, Yazeed Alshuweishi, Mohammad A. Alfhili

**Affiliations:** Department of Clinical Laboratory Sciences, College of Applied Medical Sciences, King Saud University, Riyadh 12372, Saudi Arabia

**Keywords:** lymphoma, Hodgkin, non-Hodgkin, Saudi Arabia, patterns, cancer registry, incidence, cancer trends

## Abstract

**Background:** Lymphomas account for approximately 10% of all cancer cases among the Saudi population. Even when separated, Hodgkin lymphoma (HL) and non-Hodgkin lymphoma (NHL) are in the top ten most commonly diagnosed cancers among Saudi men and women. Despite the substantial cost of HL and NHL to public health, the resources to assess their impact are insufficient. This study provides a two-decade detailed assessment of lymphoma incidence trends in the Saudi population. **Methods:** Analysis of the Saudi Cancer Registry (SCR) data for various incidence metrics from 2001 to 2020 was conducted. Joinpoint regression analysis was further performed to investigate temporal trends globally and by age group, gender, and administrative region. **Results:** HL cases grew by 174.1%, whereas NHL cases increased by only 80% for that time period. The HL overall Age-Standardized Incidence Rate (ASR) increased by 100% for both genders combined but remained unchanged for NHL. The median age at diagnosis for HL (20–30 years) and NHL (46–57 years) was lower than in many other nations. Our model identified increasing trends for HL with annual percentage changes (APCs) of 2.94% (CI: 2.2–3.7) and 3.67% (CI: 2.6–4.7) for males and females, respectively. The rise was mainly among young groups under 40. On the contrary, the NHL cohort revealed notable declining tendencies. We discovered alarming rates of HL in Saudi Arabia’s APC (2.23% for males and 3.88% for females) and ASR compared to other Western countries. Overall, the majority of the patients presented with advanced-stage disease at a younger age and with slight male predominance. **Conclusions:** The overall incidence of lymphoma (especially HL) has been rising among Saudis. Implementation of secondary and tertiary prevention measures, as well as management of modifiable risk factors, is warranted.

## 1. Introduction

Lymphoma represents a diverse group of malignant neoplasms that affect the lymphatic system [1]. It can be categorized into two main types: Hodgkin lymphoma (HL) and non-Hodgkin lymphoma (NHL). NHL is a frequently diagnosed malignancy with over 544,000 cases and around 260,000 deaths worldwide in 2020, making up 2.8% and 2.6% of the cases, respectively [2]. Furthermore, HL was the cause of 0.4% and 0.2% of all newly reported cancer-related cases and mortalities globally in that year. In the United States, NHL accounted for 4% of cases in both genders in 2023 and was the seventh and sixth most common cancer type in the US among men and women, respectively [3]. Additionally, the NHL ranked as the ninth most common cause of cancer-related deaths, accounting for 3–4% of the cases in the US that year. In Saudi Arabia, NHL accounted for 6.8% and 4.3% of cases of men and women, respectively, in 2020. As a result, NHL ranked the third most common cancer type among men and the sixth among women in the country that year [4]. Moreover, HL accounted for 5.6% and 3% of all cases that year, ranking sixth among males and seventh among females, respectively. The global burden of lymphoma is substantial. It is estimated that by 2040, the number of NHL and HL incident cases will increase to approximately 778,000 and 107,000 cases globally [5,6]. Even though lymphoma constitutes a major public health issue in Saudi Arabia, the resources for its data metrics are insufficient in contrast to several countries in the West. Therefore, it is crucial to thoroughly describe the incidence parameters of lymphoma among Saudis, taking into consideration the main variables, such as sex, age, disease subtype, dissemination of the disease, and regional distribution. This study provides a detailed assessment of lymphoma incidence metrics from 2001 to 2020. This work can positively impact the strategies for the development of policies and the establishment of early detection and screening plans into practice with the ultimate goal of lowering cancer mortality.

## 2. Materials and Methods

### 2.1. Data Collection

Five regional offices under the direction of a main office are used by the Saudi Cancer Registry (SCR) to gather information, providing comprehensive monitoring of every medical facility in the country. An examination of reports from the beginning of 2001 to the end of 2020 was performed retrospectively. Data for any year after 2020 were not yet publicly accessible when this manuscript was written. All age groups and administrative regions of Saudi male and female patients with lymphoma were studied, and relevant information, such as descriptive characteristics and other statistical parameters, was presented. The data collected covers each of Saudi Arabia’s 13 administrative regions. Cases lacking International Classification of Diseases (ICD) codes and/or with unknown nationalities were eliminated from the data analysis.

### 2.2. Statistical Parameters

All of the stated incidence statistical parameters matched the information from the SCR records. Each incidence rate was given as a number of 100,000 population.

The parameters were calculated as follows:

Age-specific incidence rate (AIR):

$$\mathrm{AIR}=\frac{\mathrm{No. of cancer cases occurring during a specific period in a population of a specific age group}}{\mathrm{No. of midyear population of that age group}}$$

Age-standardized incidence rate (ASR):

$$\mathrm{ASR}=\frac{\mathrm{Total no. of expected cases}}{\mathrm{Total standard population size}}$$

The ASR is a concise estimate of the rate that would take place in a population with a standard (reference) age structure. In this scenario, the World Standardized Population was employed as a reference.

To ascertain whether or not the data had a normal distribution, a Shapiro–Wilk normality test was executed. After normality was confirmed, a two-sided *t*-test was performed for independent two-group comparisons. A Wilcoxon rank sum (Mann–Whitney) test was performed for data without normal distribution. Ninety-five percent confidence intervals (CI) were calculated, and *p*-values were deemed significant if they were less than 0.05.

### 2.3. Temporal Trends Estimation

Temporal trends for age-standardized rates were predicted using the Joinpoint regression software (version 5.0.1 [7]), which assesses changing linear trends across a series of time intervals. In order to decrease the likelihood of undue variability in the way trends are reported over time, the models that were employed had limited joinpoints. A Z-test was used to determine if the changes were statistically distinct from zero. Annual percentage change (APC) was used, along with a 95% confidence interval (CI), and a significant level of *p*-value = 0.05 was applied to report trends. The results of this modeling analysis can be viewed as a summary of incidence rates for the selected time period. Positive values indicate increasing trends, whereas values less than zero indicate diminishing trends. This approach has widely been used to evaluate shifts in incidence patterns [8,9,10,11,12,13,14].

## 3. Results

### 3.1. Total Number of Cancer Cases in Saudi Arabia

**Figure 1:**
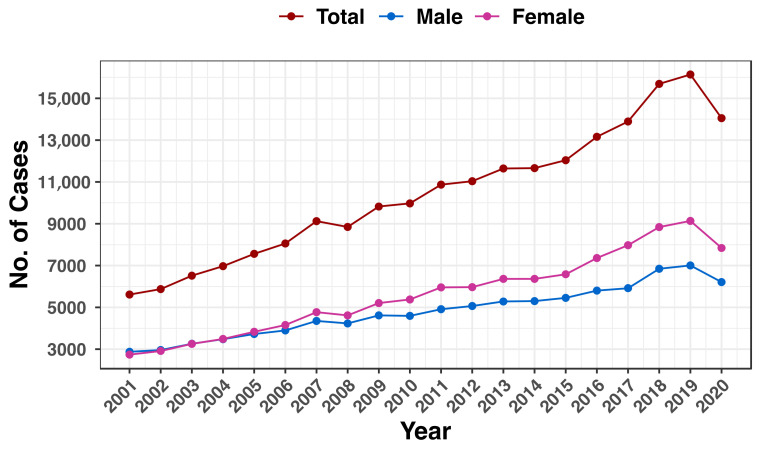
An annual overview of Saudi Arabia’s cancer cases. The total number of cancer cases for all genders (brown), men (blue), and women (pink) from 2001 to 2020 is displayed on the y-axis.

The total number of cancer cases for both genders increased by 150.2%, from 5616 in 2001 to 14,050 in 2020 (reached a peak of 16,139 cases in 2019). For males, 2875 and 6209 cases were reported throughout that period, representing a 116% rise. Females experienced a higher increase, with 2741 cases recorded in 2001 and 7841 cases in 2020, representing an 186.1% increase in the number of cancer cases overall (Figure 1).

### 3.2. The Number of Cases and Age at Lymphom Diagnosis of Lymphoma in the Saudi Population

**Figure 2:**
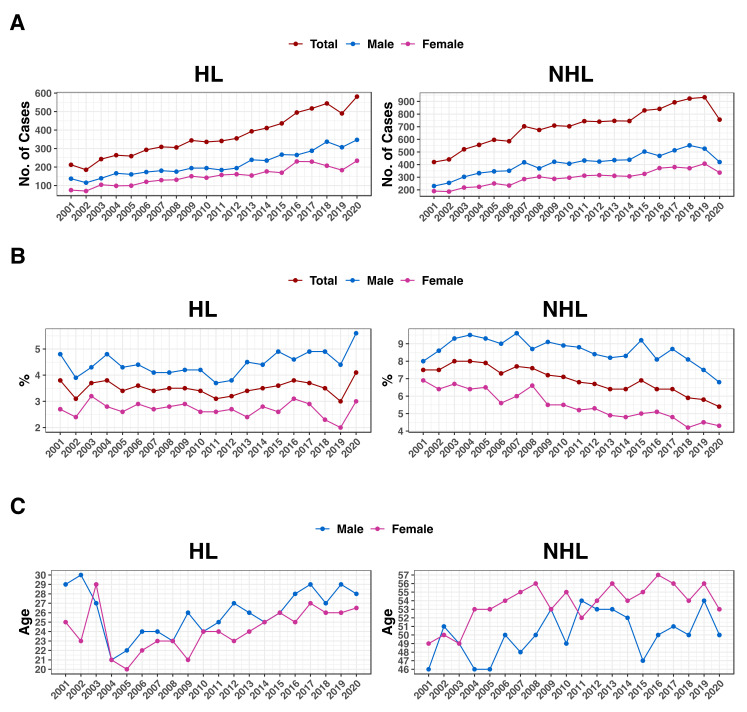
Lymphoma cases in Saudi Arabia. (**A**) The number of lymphoma cases (y-axis) among Saudis per year. (**B**) The portion of cases of lymphoma among all cases of cancer as a percentage (y-axis). (**C**) The y-axis indicates the median age of lymphoma diagnosis. The years 2001–2020 are depicted on the x-axis of all figures. Males are labeled blue, females pink, and the total is brown. HL = Hodgkin lymphoma; NHL = non-Hodgkin lymphoma.

Hodgkin lymphoma (HL) cases increased by 174.1% among Saudis, from 212 in 2001 to 581 in 2020 (Table 1 and Figure 2A). HL increased by 7.9% throughout that time period, from 3.8% in 2001 to comprising 4.1% of all malignancies in 2020 (Table 1 and Figure 2B). During the same time period, the median age at diagnosis kept fluctuating between 20 and 30 years and showed similar patterns for both genders (Figure 2C).

Non-Hodgkin lymphoma (NHL) cases grew by 80%, from 420 in 2001 to 756 in 2020 (Table 1 and Figure 2A). Even though there was an increase in the number of cases during that period, the NHL proportion of all reported cancers dropped by 28%, from 7.5% in 2001 to 5.4% in 2020 (Table 1 and Figure 2B). The median age at diagnosis varied over that time period; however, it was often greater in females than in males (49–57 and 46–54 years, respectively; Figure 2C).

### 3.3. Lymphoma Incidence Rates in the Saudi Population

**Figure 3:**
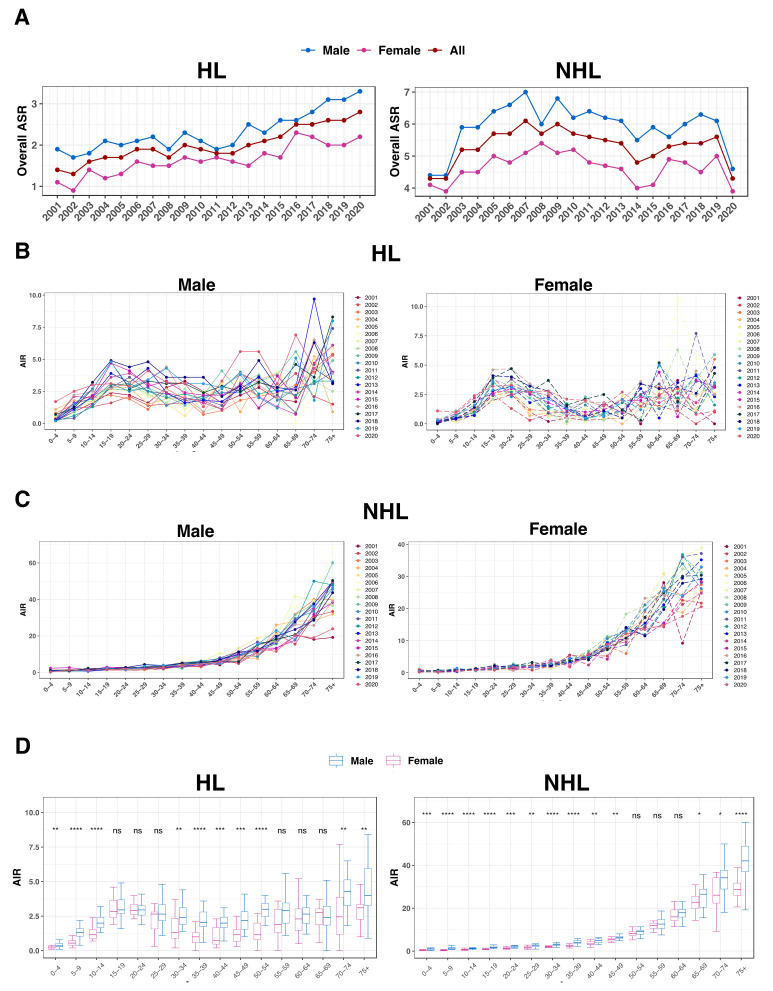
Lymphoma incidence rates in the Saudi population. (**A**) The total ASR for lymphoma across the nation for every gender (color-coded). For both genders and each age group (x-axis), AIR values are displayed for HL (**B**) and NHL (**C**). (**D**) Males (blue) and females (pink) boxplot comparisons of AIR for each age group in HL and NHL; ns denotes no significance; * *p* < 0.05, ** *p* < 0.01, *** *p* < 0.001, and **** *p* < 0.0001. Rates shown on the y-axis are expressed as per 100,000 people.

The HL overall ASR between 2001 and 2020 increased by 100% for both genders, 73.7% for males, and 100% for females (Table 1 and Figure 3A). Conversely, the ASR for NHL did not change for both genders collectively; however, it increased by 4.5% in males and decreased by 4.9% in females (Table 1 and Figure 3A).

The AIR for HL exhibited a bimodal curve with one peak that appeared at age 15–20 years and then later after the age of 50 (Figure 3B). This was not the case for NHL, where the AIR increased with age (Figure 3C). Additionally, a comparison of the AIR between both genders for each age group indicated significantly higher AIR in males in the majority of the groups, both in HL and NHL (*p* < 0.05; Figure 3D).

### 3.4. Trends in Lymphoma Incidence in Saudi Arabia

**Figure 4:**
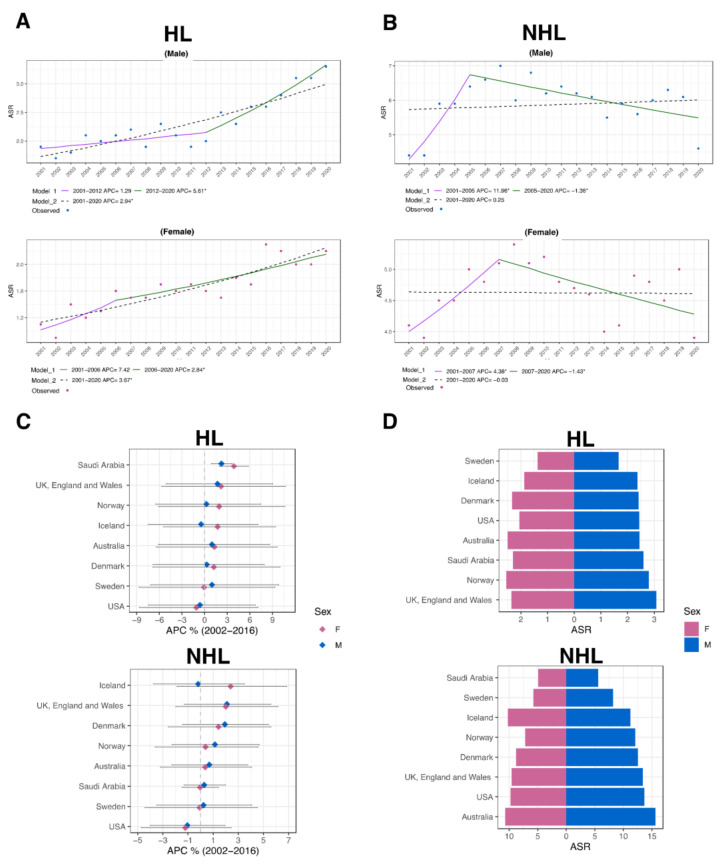
Trends in lymphoma incidence patterns in Saudi Arabia. (**A**) Temporal changes in ASR of lymphoma for both genders for HL (**A**) and NHL (**B**) between 2001 and 2020. The estimated APC for each prediction model is provided beneath each plot (* indicates *p* < 0.05). (**C**) The APC of HL and NHL ASR (2002–2016) for specified nations in comparison to Saudi Arabia. The horizontal line shows the 95% confidence interval (CI), and the central diamond reflects the estimated APC value. (**D**) A pyramid plot showing the HL and NHL ASR for both genders in Saudi Arabia and multiple nations in 2016.

A joinpoint regression analysis of the ASR to predict the trends of HL and NHL between 2001 and 2020 revealed multiple findings. In the HL group, the model exhibited growing trends for that period, with APC for males and females of 2.94% (CI: 2.2–3.7) and 3.67% (CI: 2.6–4.7), respectively (*p* < 0.05; Figure 4A). In contrast, the NHL cohort demonstrated notable declining trends, with males showing a −1.36% (CI: −2.6–−0.4) drop between 2005 and 2020 and females displaying a −1.43% (CI: −3.7–−0.4) decrease for the period between 2007 and 2020 (*p* < 0.05; Figure 4B).

Age-specific analysis of the APC in the HL group revealed multiple findings. The trends in males were significantly increasing in the age groups 25–29 (6%, CI: 4.2–7.9), 15–19 (3.9%, CI: 2.1–7.7), 30–34 (3.8%, CI: 0.9–6.7), 10–14 (3.1%, CI: 1.4–4.8), and 20–24 (2.2%, CI: 0.1–4.4). In females, the age groups with significantly increasing APC were 30–34 (10.2%, CI: 6.3–14.1), 25–29 (8.2%, CI: 3.8–12.5), 35–39 (7.5%, 1.4–13.9), 5–9 (4.6%, CI: 0.1–9.3), 20–24 (3%, CI: 0.6–5.5), and 15–19 (2.7%, CI: 0.3–5.3). The APC was less dramatic in the NHL group for both genders. In males, the only significantly increasing trend was in the 25–29 age group (3.8%, CI: 0.7–6.9), while in females, it was in the 35–39 group (2.9%, CI: 0.3–5.6). Table 2 illustrates the trend values along with 95% CI for all age groups broken down by type of lymphoma and gender.

The region-specific examination discovered multiple trends in the HL cohort. In males, the regions with significantly increasing trends were Asir (4.4%, CI: 0.4–8.7), Riyadh (2.9%, CI: 1.1–4.8), and the Eastern region (2.9%, CI: 0.8–5.1). In females, the regions with significantly increasing trends were Jouf (12.1%, CI: 6.3–18.4), Qassim (11.4%, CI: 3.4–19.9), Tabuk (8.4%, CI: 1.9–15.1), Baha (8.2%, CI: 0.4–16.6), Jazan (3.9%, CI: 0.4–7.5), Riyadh (3%, CI: 1.2–4.9), and the Eastern region (2.2%, CI: 0.2–4.3). No significant APCs were identified in the NHL cohort (Table 3).

On a global scale, computed ASR and APCs of HL and NHL for various countries were calculated for comparison with Saudi Arabia between 2002 and 2016 [15]. In Saudi Arabia, our investigation showed APCs of 2.23% (CI: 0.89–3.63, *p* < 0.05) and 3.88% (CI: 1.97–5.82, *p* < 0.05) for males and females in the HL cohort (Figure 4C). This was the highest APC compared to the other countries. In addition, an analysis of the ASR in 2016 placed Saudi Arabia among the highest three countries (Figure 4D). Although not statistically significant, the APCs for NHL were 0.29% (CI: −1.3–1.97) for males and −0.04% (CI: −1.47–1.43) for females. This is also supported by the lowest ASR in 2016 compared to other nations (Figure 4C,D).

### 3.5. Distribution of Lymphoma Stages and Subtypes

**Figure 5:**
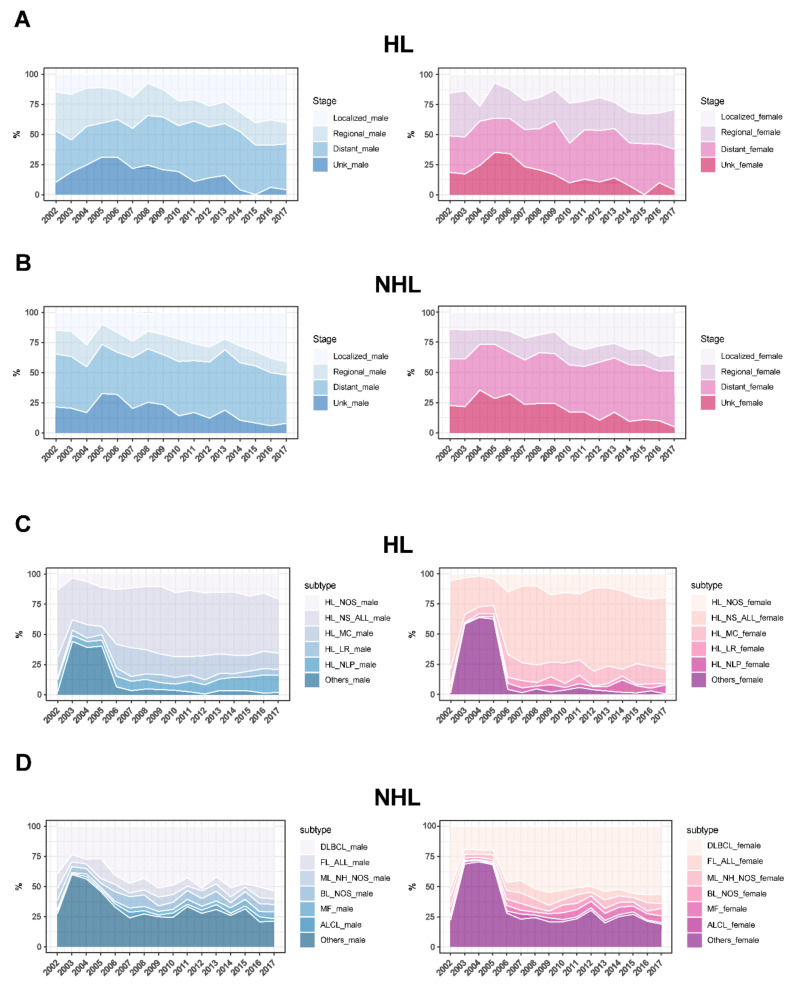
Distribution of lymphoma stages and subtypes among Saudis. HL and NHL stages (**A**,**B**) and subtypes (**C**,**D**) distributions are plotted as percentages on the y-axis in stacked area plots. For every gender, the stages and subtypes are color-coded (males = blue shades; females = pink shades). The years 2002–2017 are marked on the x-axis of all figures.

HL and NHL cases were subclassified according to stage into groups: localized, regional, distant, and unknown. Although there were fluctuations throughout the years, the area plot analysis indicated a progressive increase in the localized instances and a decrease in the unknown cases. For example, the percentage of localized male cases of HL increased from about 15% in 2002 to 29% in 2017. This pattern is similar for both the HL and NHL male and female populations. (Figure 5A,B). However, the distant stage was predominant throughout that period. In HL, the mean percentages of male and female patients with distant stages were 38.4% and 35.5%, respectively. In NHL, the percentage was 43.3% for males and 41.5% for females.

Furthermore, HL and NHL were subdivided according to the most common histological characteristics of the neoplasm. HL was subclassified into Hodgkin lymphoma, nodular sclerosis (NS), mixed cellularity (MC), lymphocyte-rich (LR), nodular lymphocyte predominance (NLP), and others. After 2006, fewer instances of unknown cases were reported, and from 2006 to 2017, the NS subtype remained consistently the most common in both males and females (median = 51.6% and 58.9%, respectively; Figure 5C). On the other hand, the NHL group was subdivided into Diffuse large B-cell lymphoma (DLBCL), follicular lymphoma (FL), Mantle cell lymphoma (ML), Burkitt lymphoma (BL), mycosis fungoides (MF), anaplastic large cell lymphoma (ALCL), and others. Similar to HL, fewer incidents of unknown cases were reported. During the period between 2006 and 2017, the DLBCL subtype was unswervingly the most commonly reported among both the NHL male (48.3%) and female (52.4%) groups (Figure 5D).

## 4. Discussion

The current study explicates the extent and direction of changes in the incidence rate of lymphoma in the Saudi population by sex, age, disease subtype, dissemination of the disease, and regional distribution from 2001 to 2020. The overall incidence of lymphoma has been rising among Saudis, especially HL, where the number of cases increased by 174.1%. In NHL, the number of cases grew by 80% for that time period. By the end of that period, HL increased by 7.9% to comprise 4.1% of all malignancies in 2020. In contrast, the NHL proportion of all reported cancers dropped by 28%, from 7.5% in 2001 to 5.4% in 2020. The evaluation of the ASR revealed similar behavior for both subtypes. The HL overall ASR increased by 100% for both genders, 73.7% for males, and 100% for females. Conversely, the ASR for NHL did not change for both genders collectively; however, it increased by 4.5% in males and decreased by 4.9% in females. The AIR for HL exhibited a bimodal curve with one peak that appeared at age 15–20 years and then later after the age of 50. This observation is not unique in our study and has been reported previously [16,17,18]. In NHL, the AIR increased consistently with age. In both subtypes, males exhibited considerably greater AIR than females for the majority of age groups.

The median age at diagnosis varied according to the lymphoma subtype. In our study, the median age at diagnosis for HL kept fluctuating between 20 and 30 years and showed similar patterns for both genders. In NHL, it was often greater in females than in males (49–57 and 46–54 years, respectively). These findings are consistent with previous studies from Saudi Arabia [18,19,20,21,22]. The median age at diagnosis for HL and NHL is younger compared to Western countries. In the West, the median age of HL diagnosis is mid-30s [23], while for NHL it is 67 [24]. The considerably younger median age of diagnosis among Saudis is not restricted to lymphoma but has also been reported in other cancer types, such as breast and colorectal cancers [13,14,25,26]. Of note, over 60 percent of the population in Saudi Arabia is under 30 years old, which could explain this trend. Other possible explanations include childhood exposure to viral agents like Epstein–Barr Virus (EBV) and Hepatitis C virus (HCV), as well as genetic, immunological, and environmental factors [27,28,29,30,31,32,33,34]. With their high consanguinity, Saudi Arabians would be a great candidate population for uncovering lymphoma susceptibility genes.

Our prediction model to anticipate the trends of HL and NHL for the period of 2001–2020 discovered multiple outcomes. The model indicated that HL trends were increasing, with annual percentage changes (APCs) for males and females of 2.94% and 3.67%, respectively. Age-specific analysis of the APC in the HL group revealed that the increase is mainly among the young groups below 40, signifying the notion of spread among younger ages. On the contrary, the NHL cohort revealed notable dropping tendencies, with males exhibiting a −1.36% drop from 2005 to 2020 and females showing a −1.43% decrease from 2007 to 2020. Our findings coincide with the universal trend of increased HL and subsided/decreased NHL incidence. In the United States, following a long history of growth, the incidence of NHL declined by approximately 1% per year from 2015 to 2019 [3]. The growing increase in NHL incidence has also been reported in multiple European countries, particularly Spain [35] and the Nordic countries [36]. Our region-specific examination discovered multiple trends in the HL cohort. The only regions with a noticeably rising tendency in both genders were Riyadh and the Eastern regions. Considering that these two regions comprise the largest portion of the kingdom and accommodate over 40% of its population, these findings are probably not surprising. Asir was male-specific, whereas Jouf, Qassim, Tabuk, Baha, and Jazan showed an increase in the female cohort. Furthermore, during our investigation over a 15-year period (2002–2016), we found alarming rates of HL in Saudi Arabia’s APC and ASR as compared to other Western countries. Saudi Arabia had the highest APC (2.23% for males and 3.88% for females) and was one of the top three countries with the highest ASR. In contrast, the APC and ASR rates for NHL were lower in comparison to other nations, which is consistent with other findings in our study.

Our analysis of HL and NHL stage distributions shows a progressive increase in localized cases and a decrease in unknown cases, suggesting an improvement in the diagnosis and reporting system. Nevertheless, the distant stage was unswervingly the predominating stage throughout that period, accounting for 35–43% of the cases depending on the type of disease and gender. The diagnosis at advanced stage is consistent with previous reports in Saudi Arabia [17,18,19,29,37]. This could be explained by multiple factors, such as the aggressive nature of the disease, negligence of the disease symptoms, or the fear of seeking medical attention. The advanced-stage disease presentations highlight a public health burden that could be alleviated. For example, the risk of HL relapse is 10–15% for early stage but 40% for advanced stage [38]. Patients with early-stage classical HL have a favorable prognosis, with very high cure rates of over 90% [39]. Moreover, treatment for the most prevalent NHL subtype (Diffuse large B-cell lymphoma) in the localized stage has a good prognosis, with complete response rates ranging from 75 to 90% [40,41,42,43].

Consistent with previous reports, nodular sclerosis (NS) and diffuse large B-cell lymphoma (DLBCL) were the most commonly reported subtypes in HL and NHL, respectively. In our analysis, the median percentages for the NS subtype were 51.6% and 58.9% for males and females, respectively. This pattern is similar to what has been reported in the US and Europe [44,45,46,47]; however, higher than in Africa and East and South Asia [48]. For NHL, the percentages for the DLBCL subtype were 48% for males and 52.4% for females. While the trend is similar, the number is rather greater than in the US and other Western countries, where it is approximately 30% [49,50].

The general distribution of HL and NHL in our study population diverges notably from several other countries. In our analysis, HL accounted for 43.5% of all lymphoma cases, whereas NHL accounted for 56.5%. According to the GLOBOCAN report for 2020, the global distribution of HL was 13%, while it was 86.8% for NHL [2]. In the US, HL and NHL accounted for 10% and 90% of lymphomas, respectively [3]. This pattern was also similar in multiple countries, such as the UK [51], Spain [52], and Canada [53]. It is known that HL patients tend to be diagnosed at a younger age compared to NHL. The increased prevalence of HL in Saudi Arabia could be explained by the high percentage of the population with younger age (60% are under 30). In addition, family history and inherited mutations are genetic risk factors for HL and NHL. It is possible that the high consanguinity among the Saudis can add more predisposing variants to HL. In fact, a large study of families with HL identified multiple predisposing loci linked to increased risk of the disease [54].

This study represents the longest and most extensive examination of lymphoma incidence patterns in the Saudi population. Nevertheless, additional information on mortality and survival rates is vitally needed. With lower NHL ASR observed in our investigation, it would be plausible that fatality rates have also fallen. In addition, information about common sites for extra-nodal spread would have been vital to understanding disease behavior. Moreover, due to the retrospective nature of data collection from numerous locations throughout the nation, bias may also be a limitation of this study. Regardless of these limitations, the study’s strengths outweigh these drawbacks and provide thorough projections of lymphoma trends in Saudi Arabia.

## 5. Conclusions

The overall incidence of lymphoma has been rising among Saudis, especially HL, which continues to comprise a proportion of all malignancies. Overall, the majority of the patients were presented with advanced-stage disease and at a younger age. Our model indicated that HL trends were increasing annually. For most age categories, males have significantly higher AIR than females. While NHL rates were low, we found alarming rates of HL in Saudi Arabia’s APC and ASR as compared to other Western countries. Therefore, secondary preventions, such as screening programs to offer early detection, coupled with efficient tertiary management of the disease post-diagnosis, are warranted. Moreover, managing modifiable risk factors such as exposure to chemicals, tobacco, infections, access to healthcare, diet, physical activity, and others needs to be a priority.

## Figures and Tables

**Table 1:** Summary of HL/NHL incidence parameters among the Saudi population.

Parameter	Sex	2001		2020		% Increase (2001–2020)	
		HL	NHL	HL	NHL	HL	NHL
Total number of cases among Saudis	Male	137.0	230.0	347.0	420.0	153.3	82.6
	Female	75.0	190.0	234.0	336.0	212.0	76.8
	Total	212.0	420.0	581.0	756.0	174.1	80.0
% of HL/NHL cases of all cancer cases among Saudis	Male	4.8	8.0	5.6	6.8	16.7	−15.0
	Female	2.7	6.9	3.0	4.3	11.1	−37.7
	Overall	3.8	7.5	4.1	5.4	7.9	−28.0
Overall ASR	Male	1.9	4.4	3.3	4.6	73.7	4.5
	Female	1.1	4.1	2.2	3.9	100.0	−4.9
	Overall	1.4	4.3	2.8	4.3	100.0	0.0

Abbreviations: ASR = overall age-standardized incidence rate.

**Table 2:** Trends in HL/NHL incidence by age and sex groups (2001–2020).

Age Group	Sex Group	APC		95% CI	
		HL	NHL	HL	NHL
0–4	M	0.97	−0.3	[−5.4–7.9]	[−5.2–4.7]
	F	1.32	−3.1	[−4.0–7.0]	[−9.5–3.8]
5–9	M	2.13	−0.1	[−1.8–6.3]	[−3.4–3.3]
	F	4.6 *	2.4	[0.1–9.3]	[−2.5–7.6]
10–14	M	3.1 *	1.0	[1.4–4.8]	[−1.6–3.7]
	F	3.6	2.7	[−0.1–7.5]	[−1.6–7.2]
15–19	M	3.9 *	2.00	[2.1–5.7]	[−0.8–4.9]
	F	2.7 *	−0.7	[0.3–5.3]	[−3.4–2.0]
20–24	M	2.2 *	1.6	[0.1–4.4]	[−0.8–4.1]
	F	3.0 *	−0.5	[0.6–5.5]	[−4.0–3.3]
25–29	M	6.0 *	3.8 *	[4.2–7.9]	[0.7–6.9]
	F	8.2 *	1.5	[3.8–12.5]	[−1.9–5.0]
30–34	M	3.8 *	1.7	[0.9–6.7]	[−0.4–3.8]
	F	10.2 *	1.6	[6.3–14.1]	[−1.0–4.1]
35–39	M	3.2	−0.2	[−0.4–6.9]	[−2.7–2.3]
	F	7.5 *	2.9 *	[1.4–13.9]	[0.3–5.6]
40–44	M	1.1	0.3	[−2.2–4.6]	[−2.3–3.1]
	F	5.5	−2.0	[−3.1–14.9]	[−5.2–1.4]
45–49	M	2.9	−0.4	[−0.5–6.3]	[−2.1–1.3]
	F	4.2	−0.9	[−1.9–10.6]	[−3.2–1.4]
50–54	M	3.1	0.4	[−0.1–6.3]	[−1.6–2.4]
	F	5.6	0.2	[−1.2–12.8]	[−2.0–2.3]
55–59	M	1.5	1.1	[−2.6–5.8]	[−1.1–3.5]
	F	3.5	0.8	[−4.6–12.4]	[−1.3–2.8]
60–64	M	−1.2	−1.3	[−4.6–2.3]	[−2.8–0.2]
	F	3.2	−0.5	[−2.6–9.3]	[−2.7–1.7]
65–69	M	5.1	−1.4	[−4.0–14.8]	[−3.4–0.7]
	F	−1.9	−1.1	[−6.8–3.3]	[−2.9–0.8]
70–74	M	1.3	−0.4	[−2.6–5.3]	[−2.6–1.8]
	F	3.4	1.2	[−5.5–12.9]	[−2.4–4.8]
75+	M	4.0	1.1	[−1.3–9.4]	[−1.3–3.6]
	F	5.0	0.4	[−3.6–14.3]	[−1.2–2]

Abbreviations: APC = annual percentage change; M = male; F = female; CI = confidence interval. * denotes *p* < 0.05.

**Table 3:** Trends in HL/NHL incidence by administrative regions and sex groups (2001–2020).

Region	Sex Group	APC		95% CI	
		HL	NHL	HL	NHL
Asir	M	4.4 *	−0.5	[0.4–8.7]	[−3.2–2.2]
	F	2.4	0.4	[−2.3–7.4]	[−2.0–2.9]
Baha	M	6.2	3.0	[−4.6–18.5]	[−2.2–8.5]
	F	8.2 *	−2.6	[0.4–16.6]	[−8.6–3.7]
East	M	2.9 *	0.1	[0.8–5.1]	[−1.2–1.5]
	F	2.2 *	0.4	[0.2–4.3]	[−1.5–2.4]
Hail	M	0.7	−1.3	[−5.0–6.8]	[−4.0–1.3]
	F	3.9	4.8	[−2.9–11.2]	[−0.3–10.1]
Jazan	M	−4.2	−0.8	[−9.6–1.3]	[−5.8–4.4]
	F	3.9 *	−0.5	[0.4–7.5]	[−3.5–2.7]
Jouf	M	4.7	−0.9	[−2.8–12.7]	[−7.6–6.4]
	F	12.1 *	−1.5	[6.3–18.4]	[−4.9–2.0]
Madinah	M	−1.0	−1.8	[−4.2–2.4]	[−4.6–1.2]
	F	0.6	−2.4	[−3.6–4.9]	[−5.1–0.3]
Makkah	M	2.8 *	−0.6	[1.1–4.4]	[−2–0.8]
	F	2.0	−0.3	[−4.3–8.5]	[−2.2–1.6]
Najran	M	4.6	1.4	[−5–15.1]	[−2.6–5.6]
	F	6.3	4.1	[−1.6–14.3]	[−1.6–10.2]
North	M	1.2	−0.6	[−5.2–8.0]	[−8–7.4]
	F	−0.9	−1.6	[−9.7–9.0]	[−11.1–8.7]
Qassim	M	3.2	1.2	[−0.7–7.3]	[−2.8–5.3]
	F	11.4 *	0.7	[3.4–19.9]	[−3.6–5.2]
Riyadh	M	2.9 *	0.2	[1.1–4.8]	[−1.1–1.5]
	F	3.0 *	0.1	[1.2–4.9]	[−1.4–1.6]
Tabuk	M	4.5	1.5	[−1.7–11.1]	[−1.7–4.8]
	F	8.4 *	−2.3	[1.9–15.1]	[−5.7–1.2]

Abbreviations: APC = annual percentage change; M = male; F = female; CI = confidence interval. * denotes *p* < 0.05.

## Data Availability

Publicly available datasets were analyzed in this study. The data can be found here: https://nhic.gov.sa (accessed on 22 August 2023).
